# Global Identification of White Lupin lncRNAs Reveals Their Role in Cluster Roots under Phosphorus Deficiency

**DOI:** 10.3390/ijms23169012

**Published:** 2022-08-12

**Authors:** Mehtab Muhammad Aslam, Muhammad Waseem, Weifeng Xu, Li Ying, Jianhua Zhang, Wei Yuan

**Affiliations:** 1 College of Agriculture, Yangzhou University, Yangzhou 225009, China; 2 State Key Laboratory of Agrobiotechnology, School of Life Sciences, The Chinese University of Hong Kong, Shatin 999077, Hong Kong; 3 Joint International Research Laboratory of Water and Nutrient in Crop, College of Resources and Environment, Fujian Agriculture and Forestry University, Fuzhou 350002, China; 4 Department of Botany, University of Narowal, Narowal 51601, Pakistan

**Keywords:** *Lupinus albus*, phosphorus deficiency, lncRNAs, mRNAs, microRNA, functional annotation

## Abstract

Phosphorus (P) deficiency heterogeneously affected plant nutritional status and physiological performance, ultimately leading to a severe yield reduction. A few putative long non-coding RNAs (lncRNAs) responding to P-starvation in the model crops *Arabidopsis thaliana* and *Oryza sativa* have been characterized. White lupin (*Lupinus albus*) is of prime importance, and is a legume with increasing agronomic value as a protein crop as it exhibits extreme tolerance to nutrient deficiency, particularly P deficiency. Despite its adapted nature to P deficiency, nothing is known about low P-induced lncRNAs in white lupin roots. To address this issue, we identified 39,840 mRNA and 2028 lncRNAs in the eight developmental stages of white lupin root (S0–S7 and lateral root, LR) grown under P deficiency. From these 2028 lncRNAs, 1564 were intergenic and 464 natural antisense intergenic transcript (NAT) lncRNAs. We further predicted six potential targets of miRNAs with twelve lncRNAs, which may regulate P-deficiency-related processes. Moreover, the weighted gene co-expression network analysis (WGCNA) revealed seven modules that were correlated with the expression pattern of lncRNAs. Gene Ontology (GO) and Kyoto Encyclopedia of Genes and Genomes (KEGG) analysis revealed 606 GO terms and 27 different pathways including signal transduction, energy synthesis, detoxification, and Pi transport. In addition, we screened 13 putative lncRNAs that showed a distinct expression pattern in each root, indicating their role in the P deficiency regulatory network. Therefore, white lupin may be a reference legume to characterize P-deficiency-responsive novel lncRNAs, which would highlight the role of lncRNAs in the regulation of plant responses to P deficiency.

## 1. Introduction

The eukaryotic genome harbors a tiny portion of coding genes (mRNAs), while a considerable portion (>90%) of non-coding RNAs (ncRNAs) are not translated into protein [1,2]. Non-coding RNAs are broadly distributed into two main categories: small ncRNAs (<200 nucleotides in length) including microRNAs, (miRNA), small interfering RNAs (siRNAs), and Piwi-interacting RNAs (piRNAs), and long ncRNAs (lncRNAs), comprising >200 nucleotides in length [3,4]. LncRNAs are a heterogeneous class of non-coding transcripts and also possess fewer exons [5,6]. These lncRNAs are further distinguished into intronic RNAs (incRNAs), natural antisense transcripts (NATs), intergenic ncRNAs (lincRNAs), and overlapping lncRNAs, which may partially overlap coding transcripts [7,8]. Advancement in next-generation sequencing (NGS) and microarray technologies, coupled with bioinformatics, have accelerated the efficient identification of lncRNAs among different plant species such as *Arabidopsis thaliana* [9], *Glycine max* [10], *Oryza sativa* [11], *Triticum aestivum* [12], *Medicago truncatula* [13], *Brassica napus* [4], and *Zea mays* [14]. Non-coding RNAs are known to play critical role in regulating the gene expression and an array of biological processes [15], including photomorphogenesis [16], fruit development [17,18], reproductive development [19], plant−pathogen interactions [20,21,22], and nutrient deficiencies responses, e.g., nitrogen [14,23,24,25] and phosphorus deficiency [10,13,26]. 

Plants require essential nutrients to maintain plant growth, yield, and nutritional quality [27]. Phosphorus (P) is one of the major essential nutrients, constituted of nucleic acids, nucleotides, and phospholipids, which play a vital role in regulating metabolism [28]. Phosphorus exists in abundance in the soil, while inorganic P (Pi) is the only form needed by plants that is acquired from the soil; therefore, agricultural crops and ecosystems face Pi starvation issues [29]. To protect plants from Pi starvation, they have evolved numerous physiological and developmental responses, known as the P starvation rescue system (PSR) [30]. The phosphorus starvation rescue system enables plants to [31] acquire Pi from the soil via modulating the root architectural system [32]. Therefore, it is of utmost urgency to understand the molecular mechanism of phosphorus use efficiency (PUE) in plants subjected to short/long-term P-starvation conditions. However, the contribution of lncRNAs to regulate P availability has been studied. In *A. thaliana*, a potential transcriptional regulator (*PHR1*) targets 104 novel lncRNAs that are responsive to Pi deficiency [33]. Another 16 potential lncRNAs act as Pi homeostasis regulators (miR399) [33], and Npc536 lncRNA was induced in the root/shoot of *A. thaliana* under P starvation and drought stress [34]. Upon Pi deprivation, *PHO2* activity was found to be suppressed due to miR399 activation, which in returned cleaved *PHO2* facilitated Pi acquisition and translocation [35,36]. Contrastingly, with ample Pi supply, another *A. thaliana* lncRNA *IPS* targets miR399, prevent its binding with *PHO2*, which abolishes the roles of phosphate transporter 1 (PHR1), thereby maintaining optimal Pi acquisition and avoiding Pi toxicity [37]. Similarly, three Pi-deficiency-induced lncRNAs (*PDILs*) have been identified in *M. truncatula*; *PDIL1* overwhelms *MtPHO2* degradation via miR399 to regulate Pi deficiency signaling and Pi transportation, while *PDIL2*/*3* facilitates Pi homeostasis [13]. Recently, two contrasting soybean genotypes, Bogao (P-sensitive) and NN94156 (P-tolerant), exhibited 4166 and 525 differentially expressed lncRNAs under varied P levels, including rich, moderate, and low P [10], indicating the involvement of lncRNAs in mitigating plant Pi-starvation responses. Accumulating evidence suggests that lncRNAs miR399, SIZI, PHO2, and PHR1 regulate Pi acquisition [37,38], which may serve as key regulators of plant responses to environmental changes. Therefore, several Pi transporters or potential lncRNAs serve as competitors that convert organic P into Pi forms, resulting in increased Pi acquisition from the soil. 

White lupin (*Lupinus albus*) has been illuminated as a model crop to disclose root physiology and adaptive responses to P deficiency [39], and develop cluster roots (CRs) in response to P deficiency [39,40]. Cluster roots enhance Pi acquisition and exudate a huge amount of carbon metabolites such as citrate and malate [40,41]. Citrate exuded upon P deficiency is known to play important role in mobilizing organic P complexes available in the soil [42], and provides evidence in support of the involvement of organic anions in P solubilization. In another study, citrate is a dominantly exuded organic acid by the white lupin mature CRs upon P deficiency [41], with a massive excretion of citrate upon aluminum exposure from the CRs of white lupin [43], suggesting the possible role of CRs in solubilizing organic P and chelating metal toxicity. Therefore, it is urgently needed to systematically identify candidate lncRNAs and their predicted biological mechanisms responsive to Pi starvation. To date, no studies have reported genome-wide identification and characterization of novel lncRNAs in white lupin responding to Pi deficiency. In this study, we identified a comprehensive set of Pi-deficiency-responsive lncRNAs from white lupin through the sequencing of nine paired-end libraries (S0–S7 and LR). Overall, our work provides abundant information about candidate lncRNAs and their regulatory pathways, and the network of Pi starvation responses in white lupin will advance our understanding in order to improve PUE in legumes and cereals crops.

## 2. Results

### 2.1. Identification and Characterization of lncRNAs in White Lupin Responsive to P Deficiency

**Figure 1:**
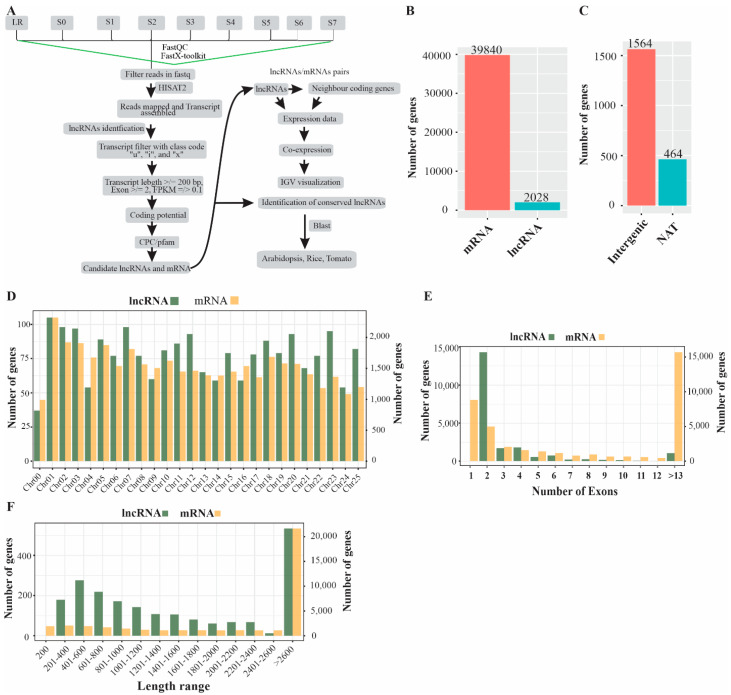
Identification pipeline and characterization of phosphorus-deficiency-responsive lncRNAs in the white lupin whole genome. (**A**) A simple pipeline to identify lncRNAs. (**B**) Number of coding (mRNA) and non-coding genes (lncRNA). (**C**) Types of lncRNA. (**D**) Chromosomal distribution of both mRNA and lncRNA. (**E**) Exon distribution of both mRNA and lncRNA. (**F**) Sequence length distribution of both mRNA and of lncRNA.

**Figure 2:**
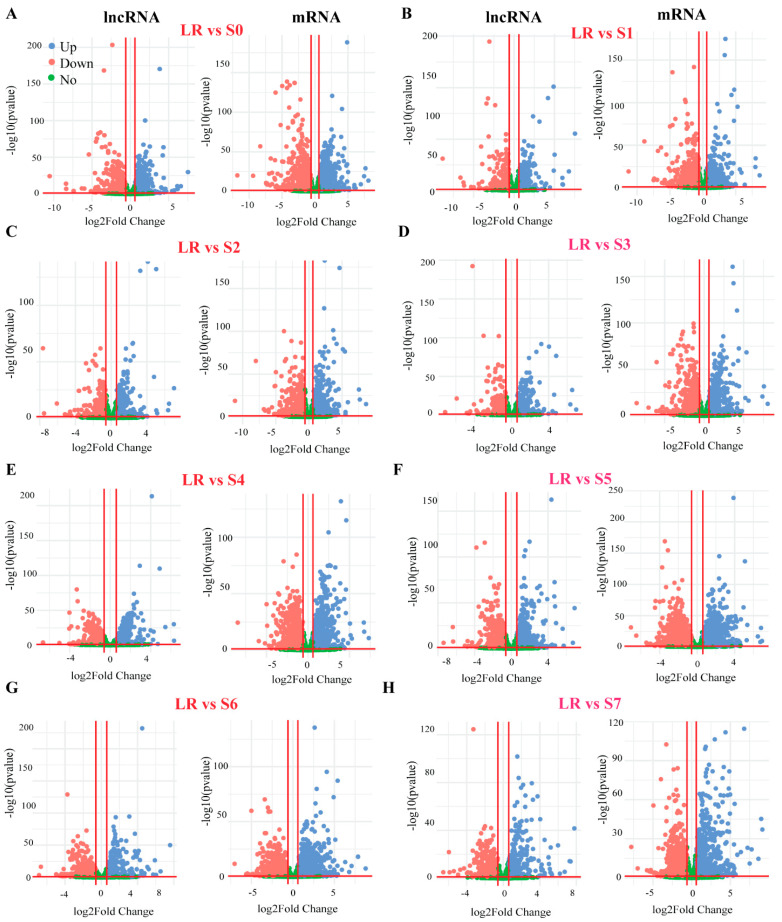
Volcano plots of differential expression (DE) analysis of white lupin roots under P deficiency. (**A**) DE genes of LR vs. S0, (**B**) LR vs. S1, (**C**) LR vs. S2, (**D**) LR vs. S3, (**E**) LR vs. S4, (**F**) LR vs. S5, (**G**) LR vs. S6, and (**H**) LR vs. S7. The left side of each panel represents lncRNA and the right side represent mRNA genes. The blue and red dot represent up and downregulation, respectively. LR, lateral root; S0–S7, different developmental stages of white lupin roots.

To identify P-deficiency-responsive lncRNAs in white lupin roots (S0–S7 and LR), we downloaded spatial RNAseq libraries from the White Lupin Genome Database https://www.whitelupin.fr/download.html, accessed on 2 January 2022. A total of nine libraries each with four replicates were sequenced with Illumina NovaSeq6000 Sequencer and 150 bp paired-end reads were generated. A pipeline was developed to identify and characterize lncRNAs in white lupin roots grown in the absence of P (Figure 1A). In this study, raw reads were quality checked and filtered before further processing. The clean reads were used to map against the white lupin reference genome and the transcripts were assembled using HISAT2. A total of 39,840 mRNA and 2028 lncRNA were identified in the whole white lupin genome (Figure 2B); the procedure applied to the prediction of lncRNAs involved their proximity class code using cuff-compare. Moreover, we obtained 1564 intergenic (class code “u”) and 464 natural antisense intergenic transcript (NAT) (class code “a”) lncRNAs (Figure 2C). Successively, we observed that lncRNAs and mRNA were unevenly distributed on all 25 chromosomes. Among the 2028 lncRNA, Chr01 contained 105, and Chr02 and Chr07 contained 98 lncRNAs. Similarly, from the 39,840 mRNA, Chr01 contained 2322, and Chr02 and Chr07 contained 1921 and 1813 mRNA, respectively (Figure 2D). In addition, we investigated the exon distribution of both the lncRNA and mRNA transcript. Most of the 4989 mRNA and 1430 lncRNA contained two exons, while 8839 mRNA and no lncRNA contained one exon (Figure 2E). A majority of lncRNA (534, 26.3%) and mRNA (21,652, 54.3%) were greater than 2600 nucleotides in length (Figure 2E). 

### 2.2. Expression of White Lupin Genes to P Deficiency 

The expression of P-deficiency-responsive genes (lncRNAs and mRNA) in the whole white lupin genome were evaluated using the fragments per kilo base of transcript per million mapped reads (FPKM) value of S0–S7 roots compared with the LR (control). The density plot of the lncRNAs expression revealed that S7 tended to exhibit a lower expression relative to the other roots (Figure S1A). We found that 553 lncRNAs were highly induced in LR, 410 lncRNAs in S7, followed by S3 (259,) S1 (258), S6 (195), and S4 (163), whereas the lowest number of lncRNAs 21 were expressed in S2 (Figure S1B). In addition, we identified that LR showed 8743 upregulated mRNA and 7164 S7, followed by S1 (7145), S5 (4486), S0 (2936), S2 (2668), and S4 (2140). The 624 and 258 mRNA were induced in S6 and S3, respectively (Figure S1C). The expression patterns of lncRNAs were distinguished from their expression patterns in the mRNAs (Figure S1B,C). Our expression data suggested that all root sections displayed the opposite expression trend between lncRNAs and mRNA. 

### 2.3. Differentially Expressed Genes (DEGs) in White Lupin Roots under P Deficiency

**Figure 3:**
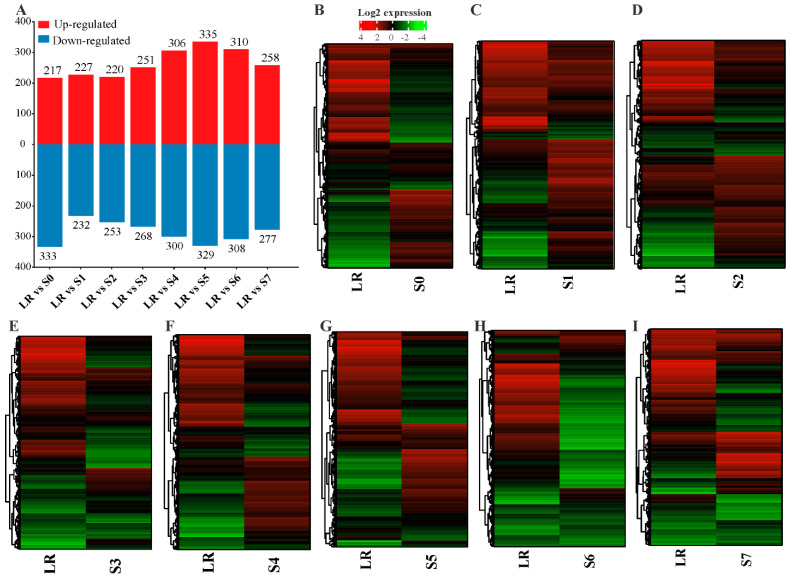
Number of differentially expressed lncRNAs (DE-lncRNAs) in different roots of white lupin. (**A**) Number of up and down-regulated genes in different root sections compared with LR, (**B**) DEGs heatmap of LR vs. S0, (**C**) LR vs. S1, (**D**) LR vs. S2, (**E**) LR vs. S3, (**F**) LR vs. S4, (**G**) LR vs. S5, (**H**) LR vs. S6, and (**I**) LR vs. S7. The red and green color represent up and down-regulation, while the black color shows no expression. LR, lateral root; S0–S7, different sections of white lupin roots.

To identify the lncRNAs and mRNA responsive to P deficiency, we identified the differentially expressed gene (DEGs) transcripts through pairwise comparisons of S0–S7 with LR under P-deficiency conditions. In total, 2028 lncRNAs and 39,840 mRNAs were screened out from the whole white lupin genome. In order to identify the transcript abundance of DE-lncRNAs and DE-mRNAs, we evaluated them using log2FoldChange. We found that LR vs. S0 showed 217 up and 333 down (Figure 2A), LR vs. S1 showed 227 up and 232 down (Figure 2B), LR vs. S2 showed 220 up and 253 down (Figure 2C), LR vs. S3 showed 251 up and 268 down (Figure 2D), LR vs. S4 showed 306 up and 300 down (Figure 2E), LR vs. S5 showed 335 up and 329 down (Figure 2F), LR vs. S6 showed 310 up and 308 down (Figure 3G), and LR vs. S7 showed 258 up and 277 down-regulated lncRNAs under P deficiency (Figure 2A,H). 

To identify the effect of P deficiency on DEGs, we identified all roots (S0–S7) compared with LR showed 5439 up and 5963 down-regulated mRNAs, except S6, which exhibited 2875 up and 3111 down-regulated mRNAs under P deficiency. In addition, to identify P-deficiency-responsive lncRNAs, we analyzed DE-lncRNAs through pairwise comparisons of S0–S7 with LR under low P conditions, and a total of 664 DE-lncRNAs were identified in the S5 root, followed by 618 (310 up and 308 down-regulated), 606 (300 up and 306 down-regulated), 550 (217 up and 333 down-regulated), 535 (258 up and 277 down-regulated), 519 (251 up and 268 down-regulated), 473 (220 up and 253 down-regulated), and 459 (227 up and 232 down-regulated) in S6, S4, S0, S7, S3, S2, and S1, respectively (Figure 3A). In some tissues, DE-lncRNAs showed a similar expression pattern such as S1, S2, and S0, S4, while other tissues behaved differently (Figure 3B–I), suggesting an aberrant expression of lncRNAs in a tissue-specific manner. In comparison with LR, more lncRNAs tended to be highly expressed in S1 and S2 root tissues (Figure 3C,D). All these observations demonstrate that lncRNAs had a higher tissue specificity in response to P limitation.

### 2.4. Weighted Gene Co-Expression Network Analysis (WGCNA) of lncRNAs

**Figure 4:**
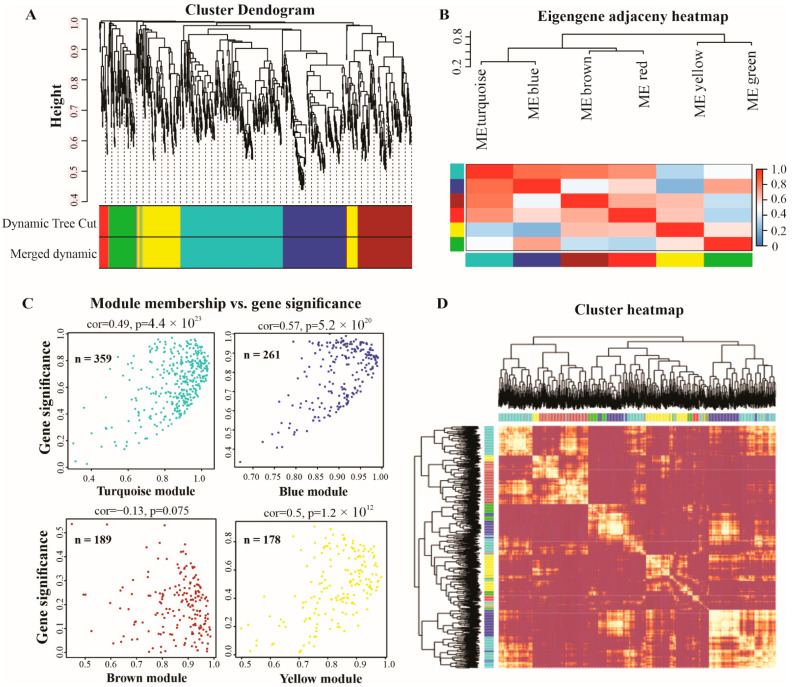
Weighted correlation network analysis (WGCNA) of lncRNAs in white lupin. (**A**) The average linkage hierarchical clustering dendogram of lncRNAs. (**B**) Modules, designated by color code, are the branches of the clustering tree. Unsupervised hierarchical clustering dendogram and heatmap. (**C**) Scattered plot of gene significance versus module membership in the most significant four modules (turquoise, blue, brown, and yellow) of lncRNAs. (**D**) Cluster heatmap representing the Topological Overlap Matrix (TOM) among all lncRNAs in the analysis. The degree of overlap is represented by the color shade; a darker color represents a higher overlap and a lighter color represents a lower overlap. The lncRNA dendogram is shown on the left, and the module assignment is shown at the top.

We performed a weighted gene co-expression network analysis (WGCNA) to construct co-expression networks of lncRNAs responsive to P deficiency. Each module represents a different color, adjusted underneath the cluster dendogram (Figure 4A). The lncRNAs show similar patterns of expression were grouped into modules and a total of seven modules were identified, with a varied number of lncRNAs—the turquoise module contained 359, blue 216, brown 189, yellow 178, green 93, red 33, and grey contained only 3 lncRNAs. The correlations among modules are depicted in Figure 4B. Thus, only turquoise, blue, brown, and yellow modules were the most significant and were selected to design the scatter plot showing each module membership versus gene significance. The turquoise module contained 359, blue 261, brown 189, and yellow 171 lncRNAs (Figure 4C). Furthermore, the eigengene adjacency heatmap indicated that these modules could be further divided into many smaller groups (Figure 4D), suggesting co-expression clusters with varying functions. 

### 2.5. Interaction Network and Functional Annotation of lncRNAs in White Lupin 

**Figure 5:**
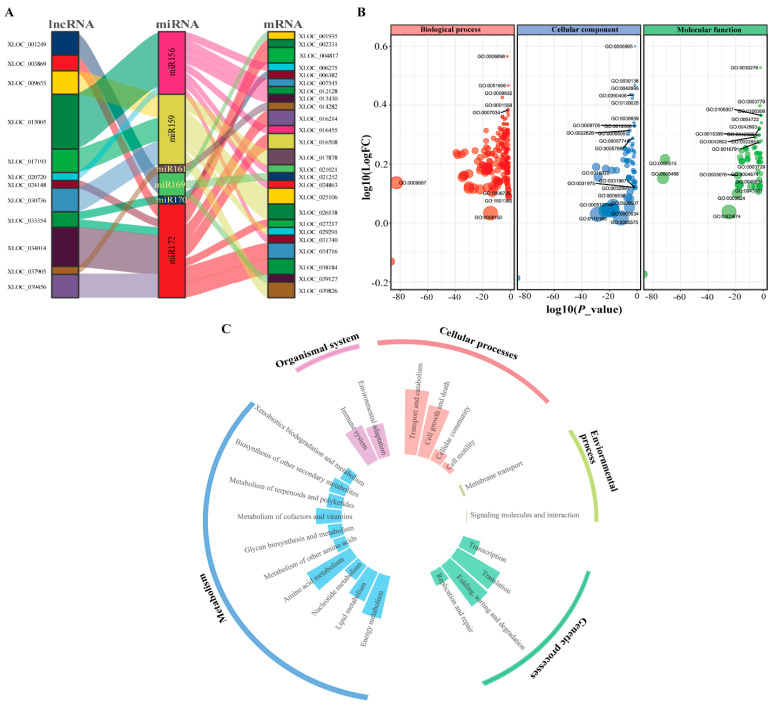
Interaction network and functional enrichment analyses of differentially expressed lncRNAs in white lupin. (**A**) Interaction network among lncRNA, miRNA, and mRNA. (**B**) Gene ontology (GO) enrichment of DE-lncRNAs targets analysis. (**C**) Kyoto Encyclopedia of Genes and Genomes (KEGG) enrichment of DE-lncRNA targets.

To reveal the interacting DE-lncRNAs in white lupin roots, we predicted the candidate targets of lncRNAs with miRNAs and mRNAs mainly harbored a potential interaction and could participate in the miRNA regulatory network. In this study, we predicted that six potential miRNAs—miR156, miR159, miR161, miR169, miR170, and miR172—were the best target of 12 lncRNAs—XLOC_001249, XLOC_003869, XLOC_009655, XLOC_015005, XLOC_017193, XLOC_020720, XLOC_024148, XLOC_030736, XLOC_033354, XLOC_034014, XLOC_037905, and XLOC_039456—corresponding to 25 mRNA genes (Figure 5A and Table S2). It is noticeable that most the mRNAs were presumably the targets of miR156 and miR172 miRNAs, suggesting that lncRNA may regulate miRNA responses to P deficiency. 

To execute the putative functions of DE-lncRNAs, we analyzed the Gene Ontology (GO) terms and Kyoto Encyclopedia of Genes and Genomes (KEGG) pathways of the putative target genes. The GO analysis of DE-lncRNAs revealed that 606 GO terms (213 in the biological process category, 330 in the molecular function category, and 63 in the cellular component category) were significantly enriched. DE-lncRNAs involved in several terms, namely, biological process including receptor-mediated endocytosis (GO0006898), detection of stimulus (GO:0051606), and regulation of cell growth (GO:0001558, GO:0007034), while cellular components, including clathrin-coated pit (GO:0005905), clathrin-coated vesicle (GO:0030136), cell projection (GO:0042995), and plasma membrane bounded cell projection (GO:0120025), and molecular functions, including clathrin binding (GO:0030276), actin binding (GO:0003779), protein serine phosphatase activity (GO:0106306), and protein serine/threonine phosphatase activity (GO:0004722), were significantly enriched (Figure 5B). Taken together, these findings suggest that DE-lncRNAs may play crucial roles in a range of biological processes under P deficiency. Subsequently, we analyzed the functional enrichment of the predicted DE-lncRNAs that underwent KEGG analysis. The targets of DE-lncRNAs were enriched in 27 KEGG pathways, including several KEGG pathways related to genetics (folding, sorting and degradation, and translation) and metabolism (amino acid and energy metabolism), indicating the role of P in producing the metabolic energy needed for plant metabolism, organismal (environmental adaptation and immune system), cellular (transport and catabolism and cell death), and environmental process (membrane transport), as shown in Figure 5C. These findings suggest that DE-lncRNAs may regulate the genes corresponding to several biological processes, including molecular and environmental adaptation, and transduction metabolism, responsive to P deficiency. 

### 2.6. Expression Profiles of Putative lncRNAs in White Lupin Roots under P Deficiency

**Figure 6:**
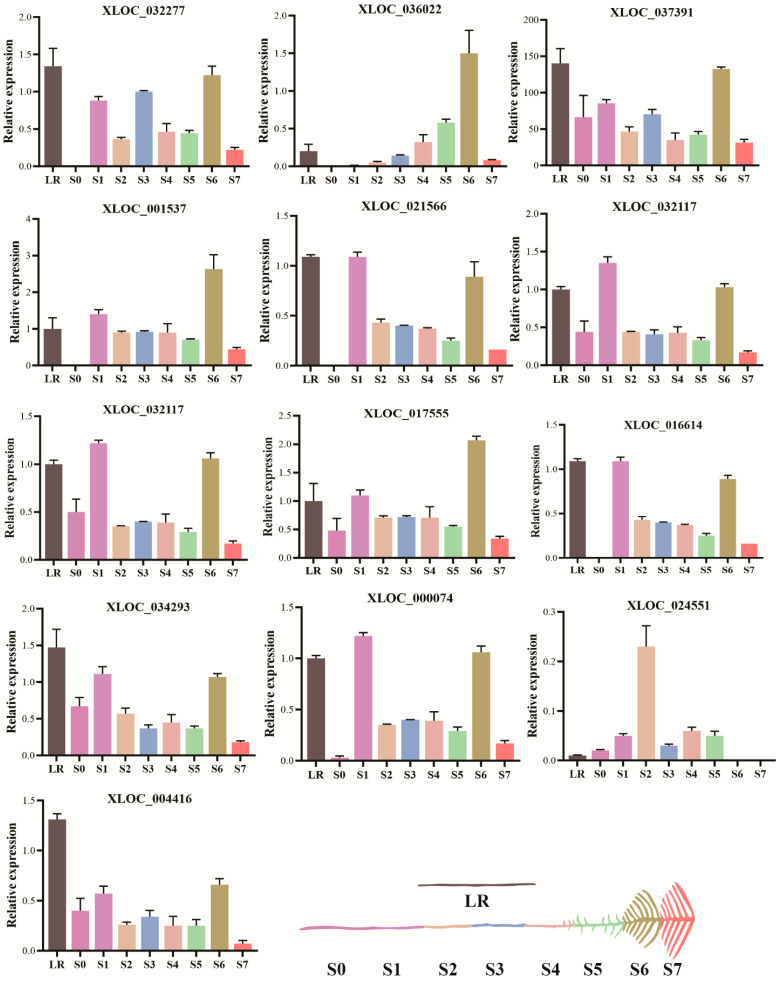
The relative expression of lncRNAs in white lupin roots responsive to P deficiency. Data represent as mean ± SE of three biological replicates. Different developmental stages of cluster roots (S0–S7) and lateral root (LR) are shown in different color.

We screened 13 putative lncRNAs in eight development stages of roots (S0–S7 and LR) and subjected to perform qRT-PCR. We found that lncRNAs presented a distinct expression pattern in each root, while most of the lncRNAs showed a higher expression in LR and least in S0 root. The S7 root showed a consistently higher expression of all lncRNAs, except XLOC_024551. In addition, there were six lncRNAs that showed an expression level in most of the roots, including XLOC_037391, XLOC_032117, XLOC_032117, XLOC_01755, XLOC_034293, and XLOC_024551. In contrast, the S6 and S7 roots showed no expression of XLOC_024551. Similarly, the S7 root showed a very low expression of most lncRNAs (Figure 6). These lncRNAs may play a role in the crop P-adaptive responses. This finding implies that potential lncRNAs are mainly involved in the P-deficiency responses, particularly LR and S6, compared with other roots in white lupin under low P stress. 

## 3. Discussion

Phosphorus deficiency is a major limiting factor affecting the agricultural industry globally [44]. Regardless of the high concentration of total soil P, only Pi can be acquired by the plant roots, and ~70% of agricultural land suffers from Pi limitation worldwide [13,45]. According to an estimation, global P reserves will begin to be depleted at the end of this century [46]. Thus, there is an urgent need for research to breed P-deficiency-tolerant crop varieties and to provide a suitable strategy to improve P acquisition from low P soils. White lupin offers a model crop species with an excellent system to evaluate P-deficiency responses over many years because of its cluster root forming nature upon P deficiency [39]. To cope with P-depleted soils, cluster roots (tertiary lateral root structures) are highly efficient in P uptake and mobilization in plants [47,48,49]. Therefore, the white lupin plant as a crop is a practical alternative to assess P acclimation responses [49], thereby ameliorating P uptake for better crop growth.

LncRNAs play critical roles in plant biological processes [36,50], particularly in plant responses to phosphorus limitation [51]. The genome-wide identification of potential lncRNAs has been reported in several plants, for example, 48,345 lncRNAs were identified in *Z. mays*, 1212 in *A. thaliana* [33], 4166 in *Glycine max* [10], and 10,785 in *M. truncatula* [13] under P-sufficient/deficient conditions, indicating their potential role in the regulation of Pi acquisition in plants. However, the roles of lncRNAs to regulate P deficiency have not been studied in white lupin. In this study, we identified 39,840 mRNA and 2028 novel lncRNAs in white lupin, including 1564 intergenic and 464 NATs lncRNAs under P deficiency (Figure 1B,C); both mRNA and lncRNAs are distributed on all 25 chromosomes and 1430 lncRNAs contained two exons, while mRNA consisted of >13 exons (Figure 1D,E). The number of lncRNAs was lower than *G. max* [10], and greater than *A. thaliana* [33], suggesting that number of lncRNAs varies among different plant species in response to particular stresses. 

To identify P-deficiency-responsive genes (lncRNAs and mRNA), the expression of S0–S7 and LR roots were analyzed. We found that a higher number of lncRNAs (553) were up-regulated in LR, and the least were found in S2 (21). Similarly, LR showed a higher number of upregulated mRNA (8743), and S3 showed less upregulated mRNAs (258) compared with the other roots, indicating that both lncRNAs and mRNA display different expression patterns. In addition, we analyzed DE-lncRNAs through pairwise comparisons of each root (S0–S7) with LR under low P conditions, and observed that the number of DE-lncRNAs identified in S5 and S6 (664 and 618) was greater than those found all other roots (Figure 2 and Figure 3), indicating that S5 and S6 were more sensitive to P limitation. Zhang et al. (2021) reported that two soybean genotypes subjected to low P stress showed a higher number of DE-lncRNAs in Bogao (P-sensitive) than that found in NN94156 (P-tolerant) genotypes, respectively. However, P deficiency-induced lncRNAs may provide a rich resource for low P responsive candidates, which would be useful for future studies. In addition, co-expression analysis by WGCNA revealed that the lncRNAs in these four modules (turquoise, blue, brown, and yellow) (Figure 4) could play important roles in mitigating P starvation, could provide a molecular basis for understanding the functions of lncRNA response to low P, and laid a foundation for further functional studies on candidate lncRNAs.

Previous studies have demonstrated that lncRNAs interact with mRNAs, which affects the translation mechanism and ultimately influences the mRNAs expression [7]. For instance, Song et al. [52] reported that *NtMYB12* (P-starvation response element) induces *NtCHS* and *NtPT2* expression, which results in increased P accumulation and ultimately enhances plant tolerance to P deficiency. It has been shown that miR399 targets lncRNAs, as well as mRNAs such as *AtFer1* (ferritin) and At5G01591.1 significantly induced upon P starvation, and their promoter encodes P1BS (Phosphate starvation response 1-Binding Sequence) motif, indicating that PHR1 regulates their expression during P deficiency [33]. In rice, osa-miR827 targets two genes, *SPX-MSF1* and *SPX-MFS2* [53], and in *A. thaliana,* ath-miR827 targets *AT1G63010* involved in Pi homeostasis and transport under P starvation [54], which serves as a potential Pi transporter. In this study, we found six potential miRNAs (miRNA156, miRNA159, miRNA161, miRNA169, miRNA170, and miRNA172) to be the best targets of lncRNA and mRNA in response to P deficiency, indicating their possible role in mitigating P deficiency issues (Figure 5A). Thus, low P-inducible miRNAs target several candidate genes, and may regulate plant developmental and metabolic processes.

The predicted roles of lncRNA shows 606 GO terms and 27 KEGG pathways that are significantly enriched (Figure 5B,C), including amino acid metabolism, transport metabolism, and environmental adaptation, which indicates that these DE-lncRNAs may regulate P-responsive genes, but this hypothesis needs further research. Similarly, miR399 and PDILs regulate plants Pi homeostasis in plants, a potential regulator to overcome P starvation stress [13,55,56,57]. The expression of long intergenic non-coding RNA (lincRNA, XLOC_026030) was significantly upregulated in rice after three days of exposure to P deficiency, indicating that this lincRNA may be involved in rice adaptation to Pi starvation [58]. 

## 4. Materials and Methods 

### 4.1. Plant Material and Growth Condition

White lupin (cv. AMIGA) wild type seeds were surface sterilized by submersion in 75% ethanol for 1 min, and then with 10% NaOCl for 15 min, and were then washed thoroughly with distilled water three times. Sterilized seeds were sandwiched between moist filter paper in Petri plates and placed in a controlled growth chamber until the seed radical emerged at 24 °C after 16/8 h of light/dark photoperiod. The germinated seeds were grown in a nutrient solution in the absence of P (P deficiency), as described by Aslam et al. [39]. The nutrient solutions were changed after every 5 days and the pH of the solution ranged between 6.0 ± 0.2 using 0.1 M HCl and 0.1 M NaOH.

### 4.2. High-Throughput Sequencing and Transcriptome Assembly

The raw data of nine RNAseq libraries were downloaded from the NCBI repository with the accession number of PRJNA575804 [59]. These libraries were constructed by dissecting the white lupin root into eight developmental stages of cluster roots (S0 to S7), and the lateral root (LR, 1 cm away from primary root) was used as the control. Paired-end sequencing (2 × 100 bp) was performed on an ILLUMINA (Illumina NovaSeq 6000) sequencer. All parts of the root samples with four independent plants grown in an aerated nutrient solution (in the absence of phosphate, P deficiency) were collected to construct each of the RNAseq libraries (four biological replicates) [59]. Trimmomatic was used to clean the adapter sequence, contaminants, and low-quality reads from the raw reads. The resulting clean reads were used for quality-checking using FastQC (http://www.bioinformatics.babraham.ac.uk/projects/fastqc/, accessed on 10 February 2022). Thereafter, the white lupin genome was indexed using HISAT2 (https://daehwankimlab.github.io/hisat2/, accessed on 10 February 2022) and then all clean reads obtained from nine libraries were aligned against the indexed genome using Cufflinks (http://cole-trapnell-lab.github.io/cufflinks/, accessed on 11 February 2022). Finally, a transcriptome of map reads was assembled using Cuffmerge and all transcripts with >1 exon and >200 bp in length were filtered using in-house-built python script. 

### 4.3. Identification and Differential Expression of lncRNAs

Protein coding transcripts were removed from the assembled transcripts using the annotated Uniprot Protein Database. The resulting filtered transcripts were used to identify putative lncRNAs, transcripts < 200 bp, and less than or equal to 1 exon were removed. Thereafter, the coding potential of filtered transcripts was calculated using the Coding Potential Calculator (CPC) and Coding-Non-Coding Index (CNCI) [60,61], and transcripts with CPC < −1 and CNCI < 0 were considered as non-coding. The differential expression (DE) analyses of all transcripts, including lncRNAs and mRNAs, were calculated using fragments per kilobase of exon per million fragments mapped (FPKM) using DESeq package at *p <* 0.05.

### 4.4. Weighted Gene Co-Expression Network Analysis (WGCNA) and Functional Annotation Analysis

Weighted gene co-expression network analysis (WGCNA) R package was used to build the weighted gene co-expression network. Differentially expressed lncRNAs (DE-lncRNAs) were screened and used for the subsequent analysis. We implemented the dynamic branch-cutting algorithm with a robust measure of interconnectedness using DynamicTreeCut and were merged dynamic using WGCNA R library; modules were defined as the branches cut off of the tree and each module was labeled with unique colors. The top four modules were selected to visualize gene significance using scattered plot. Finally, a cluster heat map was designed to generate the Topological Overlap Matrix (TOM) of the lncRNAs. The interaction network of the potential lncRNAs with miRNAs and mRNAs were generated using galluvial package in R. The Gene Ontology (GO) and Kyoto Encyclopedia of Genes and Genomes (KEGG) pathways analysis was performed using the eggnog-mapper (http://eggnog-mapper.embl.de/, accessed on 15 February 2022). The GO analysis was categorized into biological processes, cellular components, and molecular functions, while the KEGG pathways were determined using *p* < 0.05 as the cut-off criterion. 

### 4.5. qRT-PCR Analysis

To investigate the relative expression level, eight white lupin root developmental stages (S0-S7) and lateral root (LR) were used to extract RNA using an RNA Isolation Mini Kit (Omega Bio-tek, Catalogue no. R6827-00) by following the manufacturer instructions. The concentration of RNA was measured using NanoDrop Spectrophotometers (Thermo Scientific, Third Avenue, Waltham, MA, USA). To remove DNA contaminants, the RNA was treated with RNase-free DNase I (BioLabs) and then 1 μg of RNA was transcribed into the cDNA using PrimeScript™ 1st strand cDNA Synthesis Kit (TAKARA). The qRT-PCR was performed using SYBR Premix Ex Taq (TaKaRa) in a Bio-Rad CFX96 real-time PCR detection system. The ubiquitin mRNA was used as an internal control. Three independent replicates of each gene sample were used to perform the qRT-PCR, and the annealing temperature was adjusted to 57 °C with 40 amplification cycles. The qRT-PCR expression was calculated using the 2^−ΔΔCT^ method [62]. The gene and internal control primers used in this study are listed in Table S1.

## 5. Conclusions

**Figure 7:**
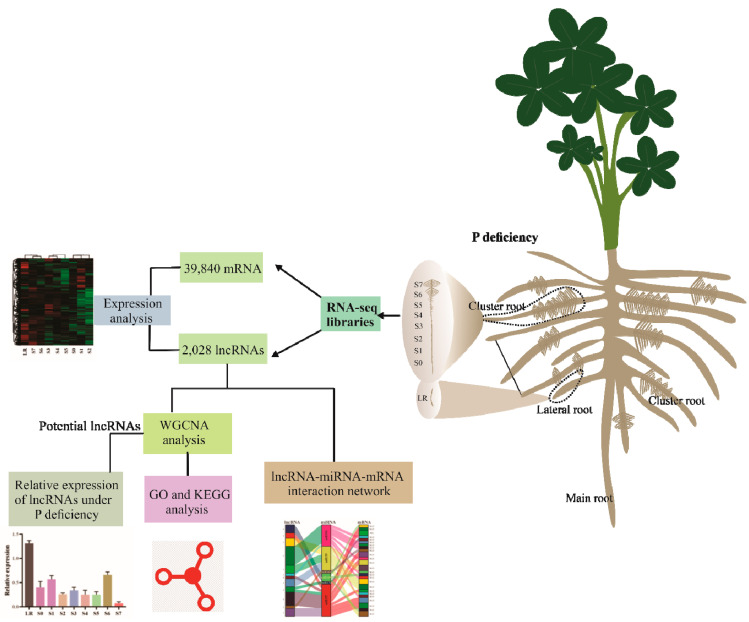
A conclusive schematic model of the findings on the identification of lncRNAs in white lupin responsive to P deficiency.

In conclusion, we identified a total of 39,840 mRNA and 2028 lncRNA, including 1564 intergenic and 464 NATs lncRNAs related to Pi starvation responses in white lupin. Further we explored the expression pattern and predicted functions of novel lncRNAs, which may provide a rich candidate resource to enhance phosphorus uptake in plants. In addition, interactions of miRNA with lncRNAs and corresponding mRNA genes related to P deficiency revealed widespread regulatory interactions between various non-coding RNAs and mRNAs (Figure 7). Our investigation provides valuable information on the global identification of novel white lupin lncRNAs and lays a foundation for further research on the means of improving P use efficiency in plants. In the future, functional-based studies on these novel lncRNAs may elucidate their specific roles in Pi starvation responses.

## Data Availability

Not applicable.

## Supplementary Materials

The supporting information can be downloaded at: https://www.mdpi.com/article/10.3390/ijms23169012/s1.
