# Supplementary material for: Global Identification of White Lupin lncRNAs Reveals Their Role in Cluster Roots under Phosphorus Deficiency
Source: Int J Mol Sci. 2022 Aug 12;23(16):9012. doi: 10.3390/ijms23169012 (PMC9409226; doi:10.3390/ijms23169012)
Supplement: Supplementary file 1 [file ijms-23-09012-s001.zip › ijms-1843077-supplementary.pdf]

International Journal of
*Molecular Sciences*

MDPI

**Table S1**. A list of primers used to perform qRT-PCR analysis.

| Gene ID | Forward Primer | Reverse Primer |
|---|---|---|
| XLOC_032277 | GTATGTCGGATAAGGATGTGATG | CCCAACAGTCCATCATTTTTAGC |
| XLOC_036022 | CATGTCAGCGAACCTTCGC | CACGAACTCATGTTGAAGTGAG |
| XLOC_037391 | TGGGCGGGATTTTCTCGC | CCGCCTCCAACTTTACGATCTC |
| XLOC_001537 | TTGTGAGCAGAATTGGAGTT | CCGTGGAGTTGAGTTGTG |
| XLOC_021566 | GCATTGAGAGGCTACCACTATGT | CTTGACTTGAGCTGATGTAAAGGAG |
| XLOC_032117 | GTTTTGGAATTAGTGGTCTCGTTT | GAAGTTAGGATGGCTGGTTCA |
| XLOC_033129 | ACCAACTATACTAAGTATTCCCTTTAGCC | ACTGAAGTCTATAGCTCAATCACACAC |
| XLOC_017555 | ATAAGATTGCTCATGCCGTGG | CTTTTCTACAATGACCACCCAAC |
| XLOC_016614 | ATCGAATTAAGCCATCAGCTCAGA | CTTGAGATGGTAGGAAAAGGTTTTGG |
| XLOC_034293 | TCGTTGGCACTGTGAGTGAG | GATTGAGGATCAAAGGAACAGGGC |
| XLOC_000074 | CTTCTGGTTTTCTTGCTTGGATG | GGCCAAAATTCAAGCCATGTA |
| XLOC_024551 | ATTTTCTGCCGTCTCTAGCCC | GTAGCGGAGTTACTGGAATGGG |
| XLOC_004416 | GGAAGAAGAGTTGATCAAAGAAGAGG | GGATCTGATGCACTCAGCTAAC |
| Ubiquitin | GTCCACACTCCACCTTGTGC | GAGGAATGCCCTCCTTGTCCT |

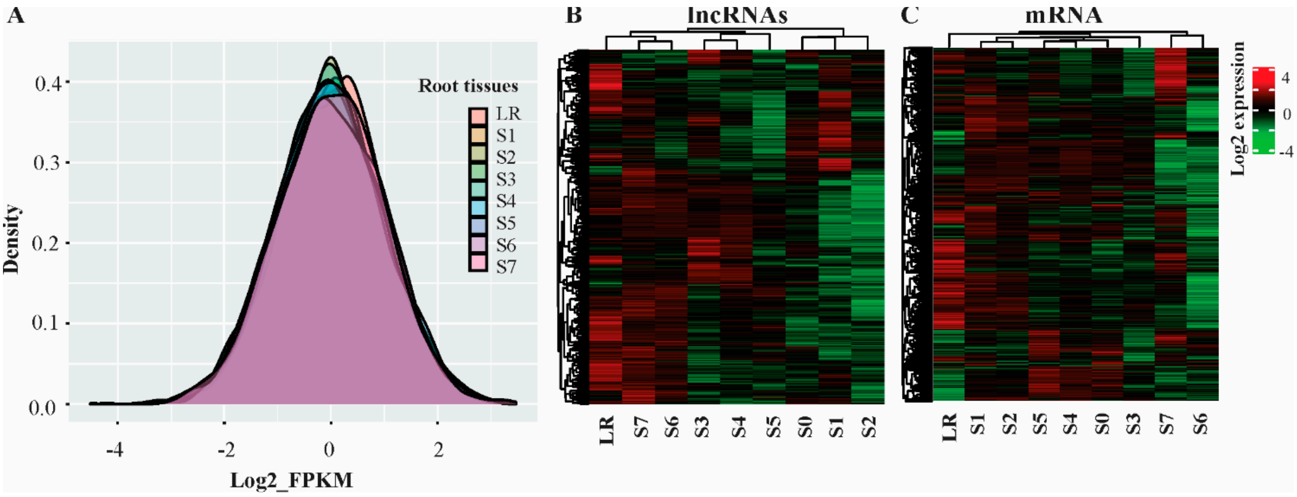


**Figure S1.** Expression of white lupin lncRNAs and mRNA in different developmental stages of roots (S0-S7 and LR) under P deficiency. **A)** Density plot of average expression of lncRNAs, **B)** Expression of lncRNAs, **C)** Expression of mRNA. S0-S7 indicates different developmental stages of cluster root and LR indicates lateral root which is used as control.
